# Combined fluorescent *in situ* hybridization for detection of microRNAs and immunofluorescent labeling for cell-type markers

**DOI:** 10.3389/fncel.2013.00160

**Published:** 2013-09-23

**Authors:** Amrita D. Chaudhuri, Sowmya V. Yelamanchili, Howard S. Fox

**Affiliations:** Department of Pharmacology and Experimental Neuroscience, University of Nebraska Medical CenterOmaha, NE, USA

**Keywords:** brain, FFPE, neuron, LNA, TSA

## Abstract

Identification of the cell type of origin for normal or aberrant gene expression is critical for many studies, and poses a significant problem for some regulatory RNAs such as microRNAs. MicroRNAs are small non-coding RNAs that regulate cellular function by targeting specific mRNAs and reducing the level of their protein product. Aberrant expression of miRNAs in cell-types where they are not normally expressed occurs in several disease conditions. Therefore, it is important to determine not only the expression level of microRNAs, but also where they are expressed. Here we describe a detailed method for fluorescent *in situ* hybridization (FISH) combined with immunofluorescent labeling for cell-type markers in formalin fixed paraffin embedded (FFPE) sections along with modifications required to adapt the protocol for primary neurons grown in culture. We have combined the specificity and stability of locked nucleic acid (LNA) probes with tyramide signal amplification. To prevent loss of small RNA species, we performed post-fixation with ethylcarbodiimide (EDC). Additionally by omitting protease digestion and using only high temperature with sodium citrate buffer for FFPE sections, we were able to perform immunolabeling for proteins concurrently with *in situ* hybridization without compromising efficacy of either procedure.

## Introduction

Since their discovery in 1993, microRNAs (miRNAs) have emerged as important regulators of cellular function. MiRNAs are synthesized as long primary transcripts (pri-miRNAs) that are processed sequentially by two RNase III enzymes, Drosha in the nucleus and Dicer in the cytoplasm, along with their associated proteins (DGCR8 and TRBP, respectively), to generate the mature form (Kim et al., 2009). These small (~22-nucleotide long) RNAs do not encode proteins. Rather they get incorporated into a protein complex, called the RNA-induced silencing complex (RISC) that regulates translation of target mRNAs. Most often, miRNAs bind to the 3′UTR of their target mRNAs and reduce the corresponding protein level by either degradation of the target or repression of translation (Filipowicz et al., 2008). One miRNA can target hundreds of mRNAs and about 60% of all protein coding genes are predicted to be regulated by miRNAs (Friedman et al., 2009). Target specificity of miRNAs is therefore often determined by their cell type and developmental stage specific expression. In the brain, for example, distinct groups of miRNAs are expressed in neurons, astrocytes, oligodendrocytes, and microglia. MiRNAs-124, 434, and 376a are specifically enriched in neurons while miRNA-143, 146a, 449a, and 193 are enriched in astrocytes (Jovicic et al., 2013). Overexpression of these neuron-enriched miRNAs can drive differentiation of neural stem cells to neurons, while overexpression of glia-enriched miRNAs prevent differentiation to neurons and promote glial differentiation (Lim et al., 2005). Aberrant expression of miRNAs in cell types where they are not expressed under physiological conditions occurs in disease states and following injury. For example, miRNA-21 and miRNA-142, two miRNAs not normally expressed in neurons, are detectable in neurons following nerve crush injury (Wu et al., 2011) and in infection-induced CNS inflammation (Yelamanchili et al., 2010; Chaudhuri et al., 2013). It is therefore important to study both the level of expression of miRNAs and their localization. Fluorescent *in situ* hybridization (FISH) is an excellent method for this purpose, as it can be combined with simultaneous immunofluorescent labeling for cell-type markers.

The earliest methods for *in situ* hybridization detection of miRNAs used digoxigenin labeled ribonucleotide probes (Chen, 2004; Kosman et al., 2004). However, due to the small size of miRNAs, the sensitivity and specificity of ribonucleotide probes is often not sufficient to distinguish true signal from background noise. A number of modifications have therefore been introduced to improve the signal to noise ratio. The first and probably most important of these modifications is the use of locked nucleic acid (LNA) probes instead of ribonucleotide probes (Koshkin et al., 1998). In LNA the 2′ oxygen and the 5′ carbon moieties of ribose are linked by a methylene bridge, locking it in a C3′-endo conformation that is the major conformation in A-form of nucleic acids and is most suitable for hybridization with RNA (Koshkin et al., 1998; Valoczi et al., 2004). This prevents denaturation of the hybridized probe and results in increased stability of the RNA-LNA hybrid, thereby increasing the melting temperature (Tm) by 2–10°C for each LNA monomer included in the probe (Koshkin et al., 1998; Valoczi et al., 2004). Additionally LNA probes have enhanced mismatch discrimination capability (Valoczi et al., 2004; Chou et al., 2005; You et al., 2006), further improving their specificity, and are comparatively resistant to degradation by nucleases (Wahlestedt et al., 2000).

One strategy used to improve sensitivity of detection is to amplify the specific *in situ* hybridization signal using the catalyzed reporter deposition method (Bobrow et al., 1989; Kerstens et al., 1995). In this method, LNA probes labeled with digoxigenin moieties on the 3′ end, 5′ end, or both, are used for hybridization. The digoxigenin moieties are recognized by horseradish peroxidase (HRP)-tagged anti-digoxigenin antibodies. Cyanine 5 (Cy5), cyanine 3 (Cy3) or fluorescein-conjugated tyramides are used as HRP substrate. The tyramides are converted by HRP to highly reactive tyramide radicals that bind covalently to nearby tyrosine residues. These radicals are extremely short-lived, preventing them from diffusing away from the site of synthesis. This technique allows approximately 500-times amplification of the original signal (Qian and Lloyd, 2003).

Recently, a number of methods have been described for combined detection of miRNA and proteins in tissue samples and cultured cells (Zaidi et al., 2000; Nuovo et al., 2009; de Planell-Saguer et al., 2010; Nuovo, 2010; Sempere et al., 2010; Schneider et al., 2011; Wu et al., 2011; Herzer et al., 2012; Nielsen and Holmstrom, 2013; Sempere and Korc, 2013). In most of these methods, immunolabeling of protein markers is performed after completion of all the FISH steps and many of them use proteinase K to break the formaldehyde cross-links and allow for better penetration of FISH probes. Here, we describe a method where IF labeling can be performed simultaneously with FISH once the probe hybridization is complete. Additionally, in our experience we found that proteinase K treatment may destroy some of the epitopes, adversely affecting the IF signal. Therefore, similar to de Planell-Saguer et al. (2010), we have eliminated the use of protease treatment and find that high heat in sodium citrate buffer, which we previously used for combined radioactive *in situ* hybridization of mRNA and colorimetric immunohistochemistry (IHC) of proteins in formalin fixed paraffin embedded (FFPE) tissue sections (Roberts et al., 2003), also sufficiently allows penetration of FISH probes for miRNA, and conserves and enhances immunoreactivity of proteins. Furthermore, to prevent loss of small RNA species during subsequent washes, we find that post-fixation with ethylcarbodiimide (EDC) as described previously (Pena et al., 2009), is beneficial. With combined use of antigen retrieval, EDC post-fixation, LNA probes, and tyramide signal amplification (TSA), we were able to simultaneously detect miRNA and cell-type markers in neurons and other cells types.

## Materials

Equipment:

1. Hybridization oven and chamber (Boekel Scientific, Feasterville, PA, USA, Catalog # 241000), or other suitable incubator/oven.

General materials required:

1. Diethylpyrocarbonate (DEPC) (Applichem, Darmstadt, Germany, Catalog # A0881).
2. TRIS (Amresco LLC, Solon, OH, USA, Catalog # 0497).
3. Sodium Chloride (Fisher Scientific, Fair Lawn, NJ, USA, Catalog # S271).
4. Hydrochloric Acid (12 M) (Fisher Scientific, Catalog # A144-212).
5. Ultrapure 20X saline-sodium citrate (SSC) (Invitrogen, Carlsbad, CA, USA, Catalog # 15557).
6. Cy 5 tyramide signal amplification (TSA) kit (Perkin Elmer, Shelton, CT USA, Catalog # NEL745001KT).
7. LNA probes (Exiqon, Woburn, MA, USA), specific for target miRNAs as well as positive and negative controls. We used snRNA U6 as positive control and a scrambled probe with no complementarity to any known miRNAs as negative control.
8. Anti-Digoxigenin-POD, Fab fragments (Roche Diagnostics, Mannheim, Germany, Catalog # 11207733910).
9. Kimwipes (Fisher Scientific, Catalog # 06-666-1A).
10. Prolong Gold anti-fade reagent with DAPI (Invitrogen, Catalog # P36935).

Materials required for deparaffinization, rehydration, and conditioning of FFPE sections:

1. Xylene (Fisher Scientific, Catalog # X5P-1GAL).
2. Ethanol 200 proof (Decon Labs Inc., King of Prussia, PA, USA, Catalog # 2701).
3. Citric acid (Fisher Scientific, Catalog # BP339-500).
4. Sodium Citrate (Fisher Scientific, Catalog # BP327-500).

Materials required for post-fixation:

1. 1-methylimidazole (Sigma-Aldrich, Saint Louis, MO, USA, Catalog # 336092).
2. N-(3-Dimethylaminopropyl)-N′-ethylcarbodiimide hydrochloride (EDC) (Sigma-Aldrich, Catalog # E1769).
3. Glycine (Sigma-Aldrich, Catalog # G7126).

Materials required for hybridization buffer:

1. Formamide (Fisher Scientific, Catalog # BP227-500).
2. AG 501-X8 Resin (Biorad, Hercules, CA, USA, Catalog # 142-6424).
3. Yeast tRNA (Invitrogen, Catalog # 15401-029).
4. Denhardt's solution 50X concentrate (Sigma-Aldrich, Catalog # D2532).
5. Ultrapure 10% SDS solution (Invitrogen, Catalog # 15553-027).
6. Ultrapure 0.5 M EDTA, pH 8.0 (Invitrogen, Catalog # 15575-020).
7. Dextran sulfate (Sigma-Aldrich, Catalog # 8906-100).

Materials required for blocking buffer:

1. Bovine serum albumin (BSA) (Sigma-Aldrich, Catalog # B4287).
2. Normal goat serum (NGS) (Vector laboratories, Burlingame, CA, USA, Catalog # S-1000).
3. Phosphate buffered saline (PBS) (Gibco, Life Technologies, Grand Island, NY, USA, Catalog # 10010).

## Buffers and solutions

Ensure that all equipment and working surfaces are RNase free. This can be done by autoclaving equipment and wiping working surfaces with RNaseZAP (Ambion, Life technologies, NY, USA, Catalog # AM9780).

The following buffers or solutions can be prepared ahead of time.

1. DEPC-treated water:
  Add 1 mL of DEPC per 1 L of water (0.1% v/v). Incubate overnight and autoclave for 30 min. Store at room temperature.
2. NaCl (3 M):
  Dissolve 87.8 g of NaCl in 500 mL of DEPC treated water.
3. Tris-HCl (1 M):
  Dissolve 121.1 g of Tris base in 800 mL of DEPC treated water. Adjust pH by adding 12 M HCl. For combined FISH and IF experiments Tris-HCl at both pH 7.4 and 8.0 need to be prepared.
4. 10X Tris Buffered Saline:
  410 ml of DEPC-treated water
  500 ml of 1 M Tris-HCl pH 7.4
  90 g of NaCl.

Note: Adding DEPC to premade Tris buffer is not recommended as DEPC forms a complex with the free amino groups of Tris. DEPC-treated water that has been autoclaved can however, be used to dissolve Tris, as DEPC is hydrolyzed during autoclaving.

5. Sodium citrate (0.1 M):
  29.41 g of sodium citrate in 1000 mL of DEPC-treated water. Store at 4°C.
6. Citric acid (0.1 M):
  9.56 g of citric acid in 500 mL of DEPC-treated water. Store at 4°C.
7. Sodium citrate buffer (0.01 M, pH = 6.4):
  Add 41 mL of 0.1 M sodium citrate to 9 mL of 0.1 M citric acid. Make the volume up to 500 mL with DEPC-treated water. Store at 4°C.
8. Hybridization Buffer:
  Deionize formamide by adding 5 g of ion exchange resin per 100 mL of formamide. Stir for 30 min at room temperature and filter through Whatman filter paper. Deionized formamide can be stored at −20°C.
  For 100 mL of hybridization buffer:
  50 mL of deionized formamide (final concentration 50%)
  1 mL of 1 M Tris-HCl, pH = 8.0 (final concentration 10 mM)
  2.5 mL of 10% SDS (final concentration 0.25%)
  200 μg/mL yeast tRNA
  1 X Denhardt's solution
  600 mM NaCl
  1 mM EDTA
  10% Dextran sulfate
  Make up to 100 mL with DEPC-treated water
  Hybridization buffer can be stored in aliquots at −20°C.
9. Blocking Buffer:
  1% BSA, 3% NGS in 1X PBS. Store at 4°C.
10. Diluted SSC:
  Dilute 20X SSC in DEPC-treated water to make 1X, 2X, and 0.2X SSC solutions.

The following buffers have to be prepared fresh on the day of the experiment.

1. Methylimidazole buffer:
  Add 1.6 ml of 1-methylimidazole to 130 ml of DEPC-treated water. Adjust pH to 8.0 by adding ~450 μl 12 M HCl, then add 16 ml 3 M NaCl and DEPC-treated water to a final volume of 160 mL. Final concentrations are 0.13 M 1–methylimidazole, 300 mM NaCl, pH 8.0.
2. EDC Solution:
  Add 307 mg EDC into 10 ml of the above methylimidazole buffer, and then readjust the pH to 8.0 by further addition of ~100 μl 12 M HCl if required. Final concentration of EDC is 0.16 M.

## Methods

Ethics Statement: Animal experiments to obtain the tissues used for these experiments were performed with approval from UNMC Institutional Animal Use and Care Committee.

For combined FISH and IF on FFPE tissue, 5 μm thick sections are cut from the tissue blocks, floated on DEPC-treated water, and picked up on glass slides and air-dried. To ensure tissue adherence slides are baked at 60°C for 1 h and cooled to room temperature the day of or the day before starting the experiment.

For combined FISH and IF on neuronal cultures, neurons grown on poly-D-lysine coated glass coverslips are fixed in 4% paraformaldehyde (PFA) for 15 min at room temperature followed by two washes with PBS for 5 min each at room temperature. Coverslips are then placed overnight in 70% ethanol at 4°C for permeabilization.

### Day 1

1.1. To deparaffinize and rehydrate FFPE sections incubate at room temperature in:

- Xylene, 3 times, 5 min each
- 100% ethanol, twice, 5 min each
- 70% ethanol (diluted in DEPC-treated water), once, 5 min
- 50% ethanol (diluted in DEPC-treated water), once, 5 min
- DEPC water, twice, 3 min each

1.2. Antigen retrieval:

Incubate slides in 0.01 M citrate buffer pH = 6.4, for 40 min at 90°C. Cool slides by incubating in citrate buffer for 20 min room temperature. Wash with TBS, 3 times, 3 min each.

Note: For combined FISH and IF on neuronal culture coverslips, the deparaffinization and antigen retrieval steps are omitted and post-fixation with EDC (next) is the first step to be performed on day 1.

1.3. EDC Treatment

- Incubate slides/coverslips in freshly prepared methylimidazole solutions, twice for 10 min each at room temperature.
- Incubate for 1 h in freshly prepared EDC solution at room temperature in a humidified chamber.
- Wash once for 5 min with 0.2% (w/v) glycine in TBS
- Wash twice, 5 min each with TBS

1.4. Prehybridization

Prepare hybridization chamber by placing 1X SSC-soaked Kimwipes at the bottom of the chamber. Place the slides or plate containing coverslips on the rack on top of the Kimwipes. Pipette enough hybridization buffer onto the tissue section so that it is completely covered. Incubate in hybridization oven at 37°C (or desired hybridization temperature) for 1 h.

1.5. Hybridization

Add 4 picomoles of 5′ and 3′ double digoxigenin-labeled LNA probe per 100 μL of hybridization buffer, mix, and heat at 65°C for 5 min to ensure denaturation of probes. Replace the hybridization buffer without probe (from pre-hybridization step) with the pre-heated hybridization buffer containing the LNA probe. Cover the tissue sections with pieces of Parafilm to prevent evaporation. Alternatively commercially available plastic slides (e.g., from IHCWorld, Woodstock, MD, USA, Catalog # IW-2601) can be used for this purpose. A thin layer of hybridization buffer will be present in between the Parafilm and tissue section and care should be taken that there are no air bubbles. For neurons grown on coverslips, the plate containing the coverslips can be sealed with Parafilm. Hybridize overnight at 37°C or optimized hybridization temperature. Preliminary experiments to optimize the hybridization temperature in order to maximize specific signal and minimize background noise may be required.

### Day 2

2.1. Stringency washes:

- Three times for 20 min each with 2X SSC at 42°C
- Twice for 20 min each with 0.2X SSC 42°C

Note: The temperature may be increased if needed to reduce background.

2.2. Blocking for IF:

Incubate in blocking buffer for 1 h at room temperature in a humidified chamber. A humidified chamber can be prepared by placing the slides on top of Kimwipes soaked in water or PBS placed at the bottom of a box with a lid.

2.3. Incubation with Primary antibodies:

Incubate with primary antibodies diluted in blocking buffer, overnight at 4°C in a humidified chamber. At this step antibodies against the desired cell-type marker can be mixed together with the anti-digoxigenin-POD. We have used up to two cell-type specific markers along with the anti-digoxigenin-POD antibody (to recognize the double digoxigenin labeled probes). The final concentration of the anti-digoxigenin-POD antibody is 1:100 in blocking buffer. Care should be taken that the antibodies for the cell-type specific markers are raised in different species. Dilutions for the cell-type specific antibodies should be determined empirically.

### Day 3

3.1. Wash the slides/coverslips twice in TBS for 2 min each at room temperature.

3.2. Incubation with secondary antibodies:

From this step onwards care should be taken to minimize exposure of the slides to light. Incubate at room temperature for 1 h with fluorochrome-labeled secondary antibodies corresponding to the species in which the primary cell-type specific antibodies were raised. We use Alexa Fluor-labeled antibodies (Invitrogen, CA, USA) for our experiments, the specific fluorochromes should match one's fluorescence filters on the microscope but not overlap with the Cy5 signal from the FISH.

3.3. Wash slides twice in TBS for 2 min each at room temperature.

3.4. FISH signal amplification with peroxidase substrate:

Dilute Cy5 standard from Cy5-TSA kit 1:100 in the provided diluent buffer. Add enough to cover tissue sections and incubate at room temperature for 10 min. Wash slides with 0.1% Tween 20 in TBS three times and twice with TBS, for 5 min each. Dip slides in DEPC-treated water before mounting in prolong gold anti-fade reagent with DAPI (Invitrogen, CA, USA). Let the slides dry at room temperature, overnight, away from light.

### Day 4

4.1. Seal slides with nail polish before imaging in a fluorescence or confocal microscope. We used a Zeiss Observer.Z1 microscope equipped with a monochromatic Axiocam MRm camera using Axiovision 40 v.4.8.0.0 software.

## Results and discussion

Several methods have been described for *in situ* hybridization of miRNAs in tissue sections (Chen, 2004; Deo et al., 2006; Nelson et al., 2006; Ryan et al., 2006; Obernosterer et al., 2007; Schaefer et al., 2007; Sempere et al., 2007; Silahtaroglu et al., 2007; Thompson et al., 2007; Williams et al., 2007; Bak et al., 2008; Nuovo, 2008; Wang et al., 2008; Lu and Tsourkas, 2009; Nelson and Wilfred, 2009; Pena et al., 2009; Yamamichi et al., 2009; Havelda, 2010; Jorgensen et al., 2010; Song et al., 2010; Soe et al., 2011; Shi et al., 2012) and a some for concurrent detection of proteins using IHC or IF (Zaidi et al., 2000; Nuovo et al., 2009; de Planell-Saguer et al., 2010; Nuovo, 2010; Sempere et al., 2010; Schneider et al., 2011; Wu et al., 2011; Herzer et al., 2012; Nielsen and Holmstrom, 2013; Sempere and Korc, 2013). Among the published methods for co-detection of miRNA and proteins in FFPE sections, Nuovo et al. described a method for colorimetric *in situ* hybridization for miRNAs using digoxigenin-labeled LNA probe (Nuovo et al., 2009). The tissue sections were digested with pepsin to facilitate penetration of the LNA probes. The recommended probe concentration was 2 picomoles/μL of hybridization buffer (i.e., 2 μM). The hybridized probe was recognized by an alkaline phosphatase (AP)-tagged anti-digoxigenin antibody and color development was performed using the AP substrate NBT/BCIP. This was followed by IHC for protein target using an automated system. The different color signals from *in situ* hybridization and IHC were converted to fluorescent signals using the Nuance system for co-expression analysis (Nuovo, 2010). This method did not allow for amplification of the hybridization signal and was therefore less effective for detection of miRNAs with low abundance in the tissues/cells of interest. Nielsen et al. and de Planell-Saguer et al. combined the advantages of LNA technology with signal amplification using TSA (de Planell-Saguer et al., 2010; Nielsen and Holmstrom, 2013). Additionally, de Planell-Saguer et al. eliminated the protease digestion step and performed antigen retrieval using high heat and citrate buffer (de Planell-Saguer et al., 2010). Sempere et al. used the TSA amplification system for detection of both the miRNA and protein of interest (Sempere et al., 2010; Sempere and Korc, 2013). They performed digestion with proteinase K to improve tissue penetration of LNA probes. After hybridization, protein labeling was performed using an automated system (Sempere et al., 2010).

We developed a method for combined FISH and IF in FFPE sections, that further improves signal to noise ratio by addition of an EDC-crosslinking step to prevent loss of small RNAs. This enabled us to use very low probe concentrations for hybridization (0.04 picomoles/μL or 40 nM) even for low abundant miRNAs, e.g., miRNA-142 in the brain. In our experience, we found that digestion of tissue sections with proteases while improving miRNA FISH signal, also resulted in loss of epitopes and worsened the IF signal. Therefore, similar to de Planell-Saguer et al. (2010) we did not perform proteinase K/pepsin digestion, but performed antigen retrieval with high heat and citrate buffer instead. We have applied this method previously for detecting miRNA localization in specific cell types in archived human brain sections (Yelamanchili et al., 2010) and brain sections from rhesus macaques (Chaudhuri et al., 2013). In this article, we have described in detail this method for combined FISH and IF.

### Optimization of hybridization temperature

**Figure 1:**
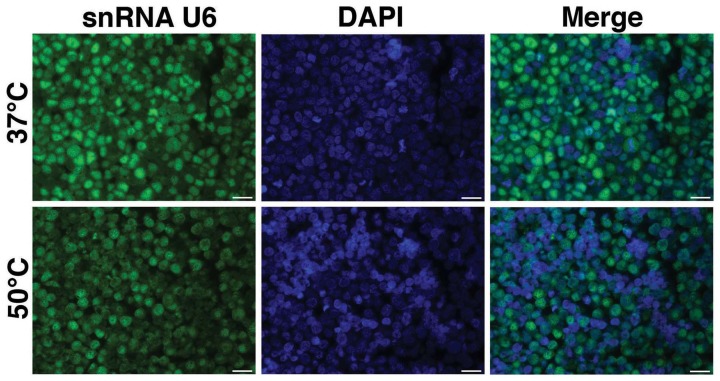
**Optimization of hybridization temperature.** FISH was performed for snRNA U6 in FFPE sections of BE(2)M17 cells. Two hybridization temperatures were compared. The snRNA U6 signal (green) appeared to be brighter in the sections that were hybridized at 37°C (top panel) compared to those that were hybridized at 50°C (bottom panel). Scale bars are 20 μm.

Exiqon provides the Tm for the LNA probes. However, this predicted Tm, may not be the same as the true Tm to immobilized miRNA in cells/sections. Tm is also influenced by components of hybridization buffers (e.g., formamide, salt). Thus, we begin with an empiric determination of hybridization temperature using hybridization at two temperatures, 37 and 50°C and/or additional temperatures. One commonly used temperature is 20°C below the provided Tm. This can be performed on controls, performing only FISH without IF. For example, examining the ubiquitously expressed snRNA U6, a positive control for miRNA FISH, on paraffin embedded BE(2)M17 cells (a neuroblastoma cell line), a much brighter FISH signal was observed at when hybridization was performed at 37°C (Figure 1).

### Post-fixation with EDC improves fish signal without affecting if signal

**Figure 2:**
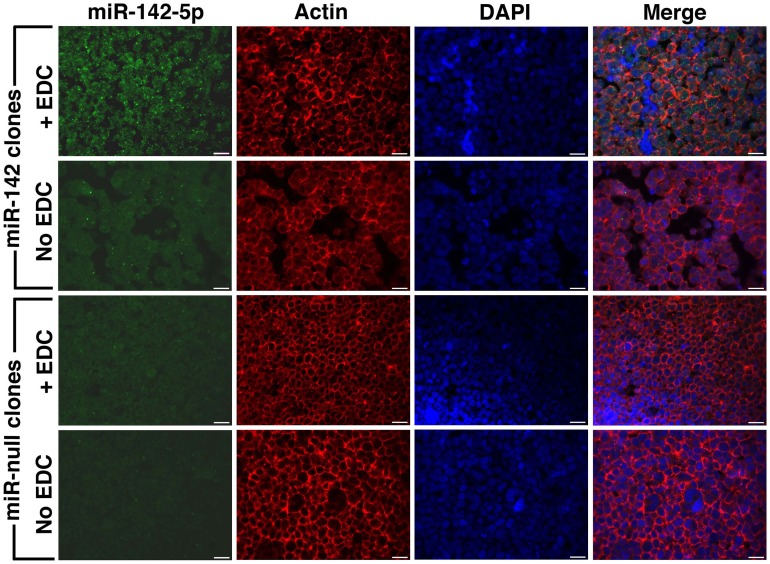
**Post-fixation with EDC improves FISH signal.** Combined FISH and IF was performed for miRNA-142-5p and actin in FFPE sections of BE(2)M17 stable clones that express miRNA-142 and those that were transfected with a control plasmid miRNA-null. After post-fixation with EDC, adequate miRNA-142-5p signal (green) could be detected in the miRNA-142 clones (top panel). Without EDC post-fixation, miRNA-142-5p signal was very low in the miRNA-142 clones. No miRNA-142-5p signal was detected in the miRNA-null clones. Scale bars are 20 μm.

EDC has been previously used in single miRNA FISH experiments to cross-link and prevent miRNA loss during the FISH procedure (Pena et al., 2009). We performed EDC post-fixation after antigen retrieval followed by combined FISH and IF for miRNA-142-5p and actin. These experiments were performed on FFPE sections of BE(2)M17 stable clones expressing miRNA-142 and clones that were transfected with the control plasmid (miRNA-null). As BE(2)M17 cells do not express any endogenous miRNA-142, these clones provided us with elegant positive and negative controls. As the miRNA-142-5p probe should show specific hybridization only in the miRNA-142 clones, any FISH signal detected in the miRNA-null clones would indicate non-specific hybridization under the conditions used. Similarly, absence of FISH signal in the miRNA-142 clones would indicate a failed hybridization. Using the combine FISH and IF protocol described, we could detect adequate miRNA-142-5p signal in the miRNA-142 clones only when sections were fixed with EDC (Figure 2). We could still detect some miRNA-142-5p signal in sections from miRNA-142 clones that were not fixed with EDC, however, the signal intensity was extremely low (Figure 2). No miRNA-142-5p signal was detected in the miRNA-null clones in either condition (Figure 2). Concurrent IF labeling for actin was performed and signal strength for actin appeared to be similar in all the sections.

### Detection of miRNA-142-5p in FFPE monkey brain sections:

**Figure 3:**
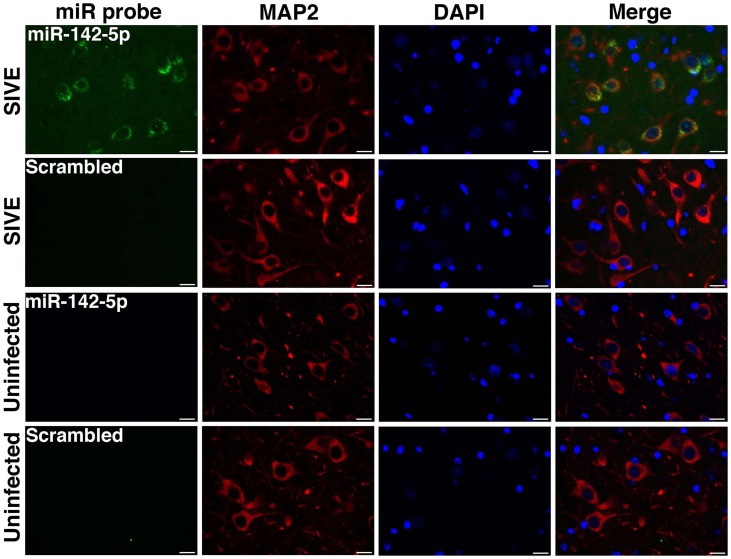
**MiRNA-142-5p is expressed in neurons in SIVE.** Combined FISH and IF was performed for miRNA-142-5p and MAP2 in FFPE hippocampal sections from rhesus macaques with SIVE and uninfected macaques. MiRNA-142-5p expression (green) was detected within MAP2-labeled neurons (red) only in sections from macaques with SIVE. No miRNA-142-5p signal was detected in uninfected control sections. A scrambled miRNA probe was used as negative control for hybridization. Scale bars are 20 μm.

**Figure 4:**
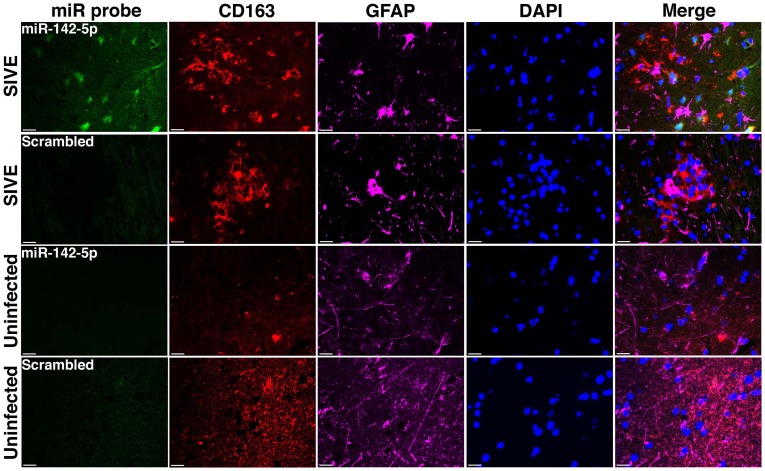
**MiRNA-142-5p is expressed in some macrophages/microglia in SIVE.** FISH was performed for miRNA-142-5p along with IF labeling for CD163 (microglia/macrophage maker) and GFAP (astrocyte marker). In cortical sections from rhesus macaques with SIVE some CD163 labeled macrophage/microglia (red) expressed miRNA-142-5p (green). No co-localization was observed for miRNA-142-5p and GFAP (magenta). No miRNA-142-5p signal was detected in uninfected control sections. A scrambled miRNA probe was used as negative control for hybridization. Scale bars are 20 μm.

We have applied the method described here to detect miRNA expression in brain sections from rhesus macaques and humans (Yelamanchili et al., 2010; Chaudhuri et al., 2013). Figures 3, 4 are representative results of such experiments. Sections of different brain regions from rhesus macaques with simian immunodeficiency virus encephalitis (SIVE) or uninfected macaques were hybridized with miRNA-142-5p probe or a scrambled miRNA probe. This was combined with concurrent labeling for microtubule-associated protein 2 (MAP2), neuron-specific marker, in hippocampal sections (Figure 3), or with astrocyte specific marker glial fibrillary acidic protein (GFAP) and microglia/macrophage specific marker CD163 (Figure 4) in cortical sections. MiRNA-142-5p expression was detected in MAP2-labeled hippocampal neurons in SIVE. In the cortical sections miRNA-142-5p was found to be expressed in some CD163 positive macrophages/microglia (Figure 4). MiRNA-142-5p signal was not detected in GFAP labeled astrocytes (Figure 4). No signal was detected in uninfected control sections as well as when the sections were hybridized with a scrambled miRNA probe.

## Conclusion

Here, we have described a method for combined *in situ* detection of miRNAs and IF labeling for cell-type markers. We modified existing methods by adding an EDC post-fixation step that greatly improved FISH signal strength without compromising IF signal. This method can be used to determine cell-types in which miRNAs are expressed in physiological and pathological conditions.

### Conflict of interest statement

The authors declare that the research was conducted in the absence of any commercial or financial relationships that could be construed as a potential conflict of interest.
